# Anesthetic Considerations for Cesarean Delivery in a Parturient With Severe Gitelman Syndrome

**DOI:** 10.7759/cureus.26260

**Published:** 2022-06-23

**Authors:** Kathleen A Smith, Monica L Reynolds, Emily H Chang, Robert A Strauss, Lacey E Straube

**Affiliations:** 1 Department of Anesthesiology, University of North Carolina School of Medicine, Chapel Hill, USA; 2 Department of Nephrology, University of North Carolina School of Medicine, Chapel Hill, USA; 3 Maternal Fetal Medicine, University of North Carolina School of Medicine, Chapel Hill, USA

**Keywords:** obstetric anesthesia, long qt syndrome, hypomagnesemia, hypokalemia, renal disease, gitelman syndrome, cesarean delivery, pregnancy

## Abstract

Gitelman syndrome is an autosomal recessive inherited disorder that impairs the function of thiazide-sensitive sodium-chloride cotransporters in the distal convoluted tubule of the nephron. During labor and delivery, avoidance of sympathetic overactivity, meticulous hemodynamic monitoring, and expedited repletion of potassium and magnesium are required to avoid adverse outcomes. We present a parturient with severe Gitelman syndrome, requiring continuous electrolyte and fluid infusions, who underwent successful cesarean delivery. Potential severe morbidity was avoided with multidisciplinary planning and management.

## Introduction

Gitelman syndrome (GS) is a salt-losing tubulopathy caused by inactivating mutations in the SLC12A3 (solute carrier family 12 member 3) gene that encodes the thiazide-sensitive sodium chloride cotransporter [1]. Reduced cotransporter activity will mimic the effects of persistent thiazide diuretic action, resulting in volume depletion, low blood pressure, and increased renin and aldosterone activity leading to renal potassium and magnesium wasting [1,2].

While GS has wide phenotypic variation, common symptoms include muscle cramping, orthostatic hypotension, palpitations, weakness, and fatigue [3]. Management includes liberal salt intake as well as potassium and magnesium supplementation. In pregnancy, expansion of extracellular volume, upregulation of the renin-angiotensin-aldosterone system, and increased renal clearance often necessitate upward titration of electrolyte supplementation [3,4].

Published data on the obstetric anesthetic management of GS is limited mostly to case reports of patients with mild disease [3,5]. We aim to increase knowledge and awareness of this rare condition and provide clinical considerations for safe cesarean delivery (CD). To our knowledge, we present the only case describing the management of a parturient with severe GS requiring continuous intravenous electrolyte replacement. Written authorization to publish this case was obtained from the patient. This manuscript adheres to the Consensus-Based Clinical Care Reporting (CARE) guidelines for publishing case reports. These guidelines can be accessed on the Enhancing the QUAlity and Transparency of Health Research (EQUATOR) website [6].

## Case presentation

We present a 24-year-old G2P0010 diagnosed with an electrolyte wasting disorder at age 10, resulting in numerous hospitalizations for syncope secondary to severe electrolyte abnormalities. Genetic testing at age 22 confirmed heterozygosity in the SLC12A3 gene for a sequence variant known to be pathogenic for GS (c.334guanine>thiamine) and a second previously unreported missense variant (c.1831adenine>cytosine). The doses of oral electrolyte supplementation she required led to intolerable gastrointestinal upset, necessitating a continuous outpatient infusion of 60 mmol/L of potassium and 205 mmol/L of magnesium at 125 mL per hour via implanted port-a-cath, originally placed in 2015 and requiring multiple replacements secondary to infection. Prior treatments, including nonsteroidal anti-inflammatory drugs (NSAIDs), spironolactone, amiloride, and eplerenone, were either ineffective or led to significant side effects. In addition to syncopal episodes, she experienced periodic generalized painful contractures and spasms several times per month following minimal exertion and requiring emergency department visits where she received large doses of fentanyl (300mcg) as well as modest doses of midazolam (2mg). Her history was significant for long QT syndrome (LQTS) secondary to electrolyte depletion, port-related thrombosis requiring life-long anticoagulation with subcutaneous enoxaparin (80mg daily), and normocytic anemia. Obstetric history included one early spontaneous abortion.

Prior to delivery, a multidisciplinary meeting involving nephrology, maternal-fetal medicine, obstetric anesthesiology, neonatology, nursing, and case management was held. Due to concern for exacerbation of hypokalemia and hypomagnesemia during labor, as well as maternal preference, a primary cesarean delivery (CD) at 36 weeks gestation was scheduled. The plan (Table 1) was provided to the patient for distribution to her care team in the event of delivery remote from our institution.

**Table 1:** Anesthetic considerations for parturients with severe Gitelman syndrome IV - intravenous access; EKG - electrocardiogram; GETA - general endotracheal anesthesia; K^+^ - potassium; Mg - magnesium; IM - intramuscular; ICU - intensive care unit

Anesthetic considerations for the management of cesarean delivery in Gitelman syndrome		
Access	Pre-induction arterial line	
	2 large bore peripheral IVs	
Monitors	Pulse oximetry	
	5-lead EKG	
	Intra-arterial blood pressure monitor	
	Defibrillation pads	
	Temperature probe	
Anesthetic choice	Combined spinal-epidural	
	If GETA required: deep anesthesia prior to intubation, laryngotracheal anesthesia	
Intraoperative considerations	Serial electrolyte monitoring: (K^+^ goal 3.0-4.5 mmol/L, Mg goal > 0.82 mmol/L)	
	Continue electrolyte infusion perioperatively	
	Active warming	
	Avoid hypotension; liberal use of fluids, vasoactive agents	
	Avoid laryngospasm, bronchospasm, hypoxia, hypercarbia, hypothermia	
	Avoid QT-prolonging drugs (ondansetron, amiodarone, volatile anesthetics, macrolides, haloperidol)	
	Torsade de pointes treatment: 30 mg/kg magnesium sulfate bolus	
	Prompt treatment of pain	
	Uterine atony management	Methylergonovine (IM)
		Misoprostol (buccal or rectal)
		Oxytocin bolus (IM/IV); avoid prophylactic infusion
		Avoid carboprost tromethamine
Postoperative considerations	Pain control: multimodal analgesia, epidural infusion, truncal blocks	
	ICU admission for electrolyte and hemodynamic monitoring	
	Perioperative telemetry	

The patient was admitted three days prior to CD for fluid and electrolyte optimization. Maintenance potassium was increased to 73 mmol/L in anticipation of increased intravascular volume associated with autotransfusion during delivery, which would likely result in hemodilution and decreased serum potassium concentration. Two units of packed red blood cells were transfused for a hemoglobin of 7.6 g/dL. She received betamethasone for fetal lung maturity. Two 18-gauge peripheral intravenous catheters were obtained; an arterial line was placed for hemodynamic monitoring and frequent blood sampling. Electrolyte infusion was continued through her port. Admission electrocardiogram revealed a QTc 459 ms. Given the potential for electrolyte aberrancy and resultant arrhythmia, telemetry was monitored perioperatively, and defibrillation pads were placed prophylactically. Anticoagulation was appropriately discontinued, and a combined spinal-epidural (CSE) was placed. Intrathecal medications included 0.75% hyperbaric bupivacaine (12 mg), fentanyl (15 mcg), and preservative-free morphine (100 mcg). Intraoperative electrolytes were monitored every thirty minutes with a goal potassium of 3.0 to 4.5 mmol/L and magnesium of greater than 0.82 mmol/L.

**Figure 1:**
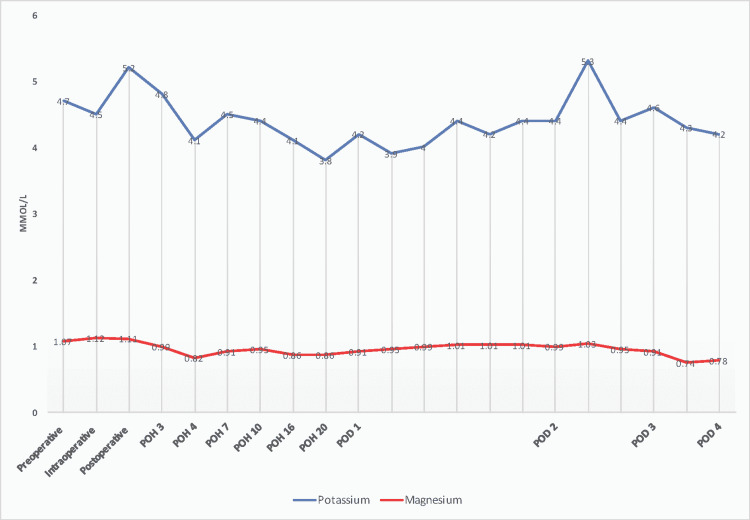
Potassium and magnesium trend throughout hospitalization POH - postoperative hour; POD - postoperative day

After delivery, uterine atony was treated with buccal misoprostol (400 mcg), intramuscular methylergonovine (200 mcg), and intramuscular oxytocin (10 units). Quantitative blood loss (QBL) was 1200 ml. The infant weighed 2500 g with initial Apgar scores of 8 and 8. Twenty minutes after delivery, he was noted to have increased respiratory effort. He was ultimately diagnosed with pulmonary insufficiency secondary to prematurity and admitted to the neonatal intensive care unit overnight but never required respiratory support. An umbilical cord blood gas was not obtained at delivery. Postoperatively, the patient was transferred to the medical ICU for close hemodynamic and electrolyte monitoring (Figure 1) and treatment. The patient was discharged on postoperative day (POD) four with electrolyte infusions at pre-admission concentrations. The pain was well-controlled with scheduled acetaminophen and as-needed oxycodone (5-10mg every six hours).

## Discussion

Although GS in pregnancy is reported, severe cases requiring continuous intravenous electrolyte repletion are rare, with little guidance regarding anesthesia care [5,7-8]. In a recently published cohort study of 43 patients, only three required IV electrolyte supplementation [3].  While parturients in this cohort had relatively mild disease and subsequent uneventful pregnancies, adverse maternal and fetal outcomes among GS patients have been reported. These are most commonly third-trimester oligohydramnios, intrauterine growth restriction, and spontaneous abortion [3,9]. The tenuous electrolyte balance and hazards associated with common anesthetics in women with severe GS obligate multidisciplinary planning for safe delivery.

LQTS is a common comorbidity in patients with GS. While our patient’s QTc was at the upper limit of normal, patients with borderline LQTS may have repolarization abnormalities unmasked by perioperative events, such as electrolyte abnormalities, including hypokalemia and hypomagnesemia, medications and hypothermia [10-12]. Given our patient’s history of syncope and LQTS due to substantial urinary losses of potassium and magnesium, she was deemed at high risk for developing a peripartum ventricular arrhythmia, including torsades de pointes and cardiac arrest [7,9,13].

In addition to prolongation of cardiac myocyte action potentials, hypokalemia and hypomagnesemia may precipitate laryngospasm, stridor, paresthesia, coma, and convulsions [10]. Administration of electrolyte-depleting medications such as diuretics and aminoglycosides and QT interval-prolonging agents (Table 2), including volatile anesthetics, amiodarone, ondansetron, macrolide antibiotics (azithromycin), and haloperidol should be avoided [10,11]. In addition to continuous EKG monitoring, defibrillator pads were applied preoperatively to allow for expeditious therapy of malignant arrhythmia. Serial potassium and magnesium measurements (every 30 minutes) were obtained using arterial line sampling (Figure 1).

**Table 2:** Common perioperative medications associated with prolonged QT interval [10,11]

Category	Examples
Inhaled anesthetics	Volatile anesthetics (sevoflurane)
Anti-emetics	5-HT3 serotonin-receptor antagonist (ondansetron)
Antihistamines	Diphenhydramine
Antibiotics	Quinolones (ciprofloxacin, levofloxacin)
	Macrolides (azithromycin, erythromycin)
Anti-hypertensives	Nicardipine
Vasoactive medications/Inotropes	Ephedrine
	Dopamine
	Isoproterenol
	Dobutamine
	Epinephrine
	Norepinephrine
	Phenylephrine
Anti-arrhythmics	Amiodarone
Anti-psychotics	Haloperidol
	Droperidol
Bronchodilators	Albuterol
	Terbutaline
Antacids	H2-receptor antagonist (famotidine)
Uterotonics	Oxytocin

Increased sympathetic tone can also precipitate malignant arrhythmias in patients with LQTS. General endotracheal anesthesia (GETA) may, therefore, pose significant risks in GS. Increases in sympathetic tone elicited by laryngoscopy, intubation, and extubation [11], as well as pain and anxiety [14] can contribute to dysrhythmias. Sympathetic response can be blunted by esmolol or remifentanil, local anesthetic topicalization of the vocal cords, and ensuring a deep level of anesthesia prior to intubation [11]. Hypercarbia, hypoxia and hypothermia may increase sympathetic tone, resulting in QT interval prolongation. Patients should be actively warmed and ventilation and oxygenation optimized [11].

Our patient had significant electrolyte wasting with minimal exertion or stress, resulting in severe contractures. Intraoperative laryngospasm, pain, or ventilator dyssynchrony could potentiate electrolyte aberrancies. We aimed to avoid general endotracheal anesthesia (GETA) unless absolutely necessary. A combined spinal epidural (CSE) was chosen over a spinal to decrease the risk of conversion to GETA in the event of insufficient spinal longevity.

GS patients are prone to hypotension from reduced vascular tone that may be poorly responsive to vasoactive agents [15]. Maintenance electrolyte-containing fluid was continued at 125 ml/hour. Given the risk of QT prolongation with all commonly used vasoconstrictors, options for prevention of spinal induced hypotension were limited. Prophylactic phenylephrine infusion was initiated at the time of spinal injection, with close observation for arrhythmia and significant bradycardia, and titrated off rapidly. No additional vasoactive medications were required.

Standard strategies for uterine atony prevention and treatment were altered to mitigate electrolyte aberrations. Oxytocin’s antidiuretic property can lead to free water retention and hyponatremia. However, a single injection does not produce the same antidiuretic effect as prolonged infusions [16]. Oxytocin can also lead to decreased vascular resistance and subsequent hypotension, a condition already common in GS [15]. Large doses of oxytocin can also prolong the QT interval. We avoided our standard prophylactic infusion (250 mu/min of 30u/500ml premade solution) and administered a single dose of intramuscular oxytocin (10 units). In addition, buccal misoprostol (400 mcg) and intramuscular methylergonovine (200 mcg) were administered for persistent uterine atony. To avoid bronchospasm and the potential for accompanying sympathetic stimulation, electrolyte imbalance and arrhythmia, carboprost tromethamine was not administered.

## Conclusions

While rare, severe GS is a high-risk maternal condition requiring providers to have disease-specific knowledge. Anticipatory delivery planning and shared decision-making with the patient that establishes a preferred mode of delivery and anesthesia management is prudent. Multidisciplinary planning allowed for detailed decision-making to occur with all involved parties, including the patient. Anesthetic goals include vigilance in monitoring and maintaining electrolyte levels within goal parameters. Care should be taken to avoid hypoxia, hypercarbia, hypothermia, pain, and anxiety, which could precipitate electrolyte aberrancy and arrhythmia. A scheduled CD in a controlled setting may be the best way to accomplish these goals. Unless contraindicated, neuraxial anesthesia with planned avoidance of medications known to prolong the QT interval is preferred.

## References

[1] Blanchard A, Bockenhauer D, Bolignano D (2017). Gitelman syndrome: consensus and guidance from a Kidney Disease: Improving Global Outcomes (KDIGO) Controversies Conference. Kidney Int.

[2] Nijenhuis T, Vallon V, van der Kemp AW, Loffing J, Hoenderop JG, Bindels RJ (2005). Enhanced passive Ca2+ reabsorption and reduced Mg2+ channel abundance explains thiazide-induced hypocalciuria and hypomagnesemia. J Clin Invest.

[3] Zhang L, Peng X, Zhao B (2020). Clinical and laboratory features of female Gitelman syndrome and the pregnancy outcomes in a Chinese cohort. Nephrology (Carlton).

[4] Cheung KL, Lafayette RA (2013). Renal physiology of pregnancy. Adv Chronic Kidney Dis.

[5] Farmer JD, Vasdev GM, Martin DP (2012). Perioperative considerations in patients with Gitelman syndrome: a case series. J Clin Anesth.

[6] (2022). The CARE guidelines: consensus-based clinical case reporting guideline development. https://www.equator-network.org/reporting-guidelines/care/.

[7] Scognamiglio R, Negut C, Calò LA (2007). Aborted sudden cardiac death in two patients with Bartter's/Gitelman's syndromes. Clin Nephrol.

[8] Venugopalan S, Puthenveettil N, Rajan S, Paul J (2020). Anaesthesia for emergency caesarean section in a patient with Gitelman syndrome. Indian J Anaesth.

[9] Calò LA, Caielli P (2012). Gitelman's syndrome and pregnancy: new potential pathophysiological influencing factors, therapeutic approach and materno-fetal outcome. J Matern Fetal Neonatal Med.

[10] Fazio G, Vernuccio F, Grutta G, Re GL (2013). Drugs to be avoided in patients with long QT syndrome: focus on the anaesthesiological management. World J Cardiol.

[11] Booker PD, Whyte SD, Ladusans EJ (2003). Long QT syndrome and anaesthesia. Br J Anaesth.

[12] Whyte SD, Nathan A, Myers D (2014). The safety of modern anesthesia for children with long QT syndrome. Anesth Analg.

[13] Bettinelli A, Tosetto C, Colussi G, Tommasini G, Edefonti A, Bianchetti MG (2002). Electrocardiogram with prolonged QT interval in Gitelman disease. Kidney Int.

[14] O'Hare M, Maldonado Y, Munro J, Ackerman MJ, Ramakrishna H, Sorajja D (2018). Perioperative management of patients with congenital or acquired disorders of the QT interval. Br J Anaesth.

[15] Calò LA (2006). Vascular tone control in humans: insights from studies in Bartter's/Gitelman's syndromes. Kidney Int.

[16] Gupta DR, Cohen NH (1972). Oxytocin, "salting out," and water intoxication. JAMA.

